# Compton imaging with ^99m^Tc for human imaging

**DOI:** 10.1038/s41598-019-49130-z

**Published:** 2019-09-09

**Authors:** Makoto Sakai, Yoshiki Kubota, Raj Kumar Parajuli, Mikiko Kikuchi, Kazuo Arakawa, Takashi Nakano

**Affiliations:** 1 Gunma University Heavy Ion Medical Center, Graduate School of Medicine, Gunma University, 3-39-22 Showa-machi, Maebashi, Gunma Japan; 2 Department of Molecular Imaging and Theranostics, National Institutes for Quantum and Radiological Science and Technology, Anagawa 4-9-1, Inage, Chiba Japan; 3 Department of Radiation Oncology, Gunma University Graduate School of Medicine, 3-39-22 Showa-machi, Maebashi, Gunma Japan

**Keywords:** Molecular medicine, Biomedical engineering

## Abstract

We have been developing a medical imaging system using a Compton camera and demonstrated the imaging ability of Compton camera for ^99m^Tc-DMSA accumulated in rat kidneys. In this study, we performed imaging experiments using a human body phantom to confirm its applicability to human imaging. Preliminary simulations were conducted using a digital phantom with varying activity ratios between the kidney and body trunk regions. Gamma rays (141 keV) were generated and detected by a Compton camera based on a silicon and cadmium telluride (Si/CdTe) detector. Compton images were reconstructed with the list mode median root prior expectation maximization method. The appropriate number of iterations of the condition was confirmed through simulations. The reconstructed Compton images revealed two bright points in the kidney regions. Furthermore, the numerical value calculated by integrating pixel values inside the region of interest correlated well with the activity of the kidney regions. Finally, experimental studies were conducted to ascertain whether the results of the simulation studies could be reproduced. The kidneys could be successfully visualised. In conclusion, considering that the conditions in this study agree with those of typical human bodies and imaginable experimental setup, the Si/CdTe Compton camera has a high probability of success in human imaging. In addition, our results indicate the capability of (semi-) quantitative analysis using Compton images.

## Introduction

A Compton camera is an imaging device that has been developed for astronomy^1,2^, beam monitoring for hadron therapy^3–5^, and environmental radiation measurements^6^. It can detect the direction of gamma rays emitted by radioisotopes (RI) based on the kinematics of Compton scattering. A fundamental Compton camera consists of two types of sub-detectors. In a Compton camera, for an individual gamma emission, Compton scattering occurs in the first detector (scatterer) and photo-absorption occurs in the second detector (absorber), which are termed a Compton event. Both the detectors (scatterer and absorber) record the interaction positions and deposited energies. When the electron is assumed to be free and at rest, the scattered angle θ in the scatterer can be calculated as

1 $$\cos \,{\rm{\theta }}=1-\frac{{{\rm{m}}}_{{\rm{e}}}{{\rm{c}}}^{2}{{\rm{E}}}_{1}}{{{\rm{E}}}_{2}({{\rm{E}}}_{1}+{{\rm{E}}}_{2})},$$

where m_e_c^2^ is the mass energy of an electron, E_1_ is the energy of the recoiled electron in the scatterer, and E_2_ is the energy deposited in the absorber. The direction of the incident gamma ray is restricted within a cone, called the Compton cone.

The application of Compton camera in nuclear medicine was first proposed by Todd *et al*.^7^. Because a Compton camera does not require a mechanical collimator, simultaneous imaging of multi-radionuclides is possible within a wide field of view (FOV), with high efficiency and across a wide energy range (several tens of keV to a few MeV). Thus, Compton cameras are a promising tool in medical imaging^8,9^.

For nuclear medical imaging, Anger cameras are widely used in the vast majority of the imaging system. Anger cameras have been explored throughout the last sixty years and used in various applications^10^. However, because of some limitations in the ability of the collimator, it is difficult to use for high energy gamma rays. On the other hand, the Compton camera can detect annihilation gamma ray emissions from a positron emission tomography (PET) agent. In addition, Fontana *et al*. proposed the utilization of higher energy gamma rays using the Compton camera^8^.

We have been developing a semiconductor-based Compton camera, originally developed by the Japan Aerospace eXploration Agency (JAXA)^11–13^. A Si semiconductor detector with low noise was adapted to our Compton camera, allowing highly accurate imaging of low-energy gamma emitters^14^. Because Si semiconductors have high energy resolution and smaller Doppler broadening than other detectors, they are suitable for low energy gamma rays as a scatterer of a Compton camera^15^.

A Compton camera aimed for medical system must be capable of imaging existing probes because the modality has been well established. For example, ^99m^Tc is the most widely used radionuclide in nuclear medical imaging because of its suitable characteristics (such as energy, half-life, and chemical ability). Some groups succeeded in imaging Tc-99m^16,17^, and we have already demonstrated the imaging ability of the Compton camera for ^99m^Tc-DMSA accumulated in rat kidneys and verified its potential for imaging on the human body^13^. At present, though the ability of our Compton camera is not superior to the ordinary Anger cameras, we aim to extend its application to human imaging. To evaluate the capability, the scaling effect and the effect of scattering of the gamma ray in the body trunk have to be considered. In this study, we performed an imaging experiment with a human body phantom to confirm the likelihood of human imaging.

## Results

### Simulation study

**Figure 1:**
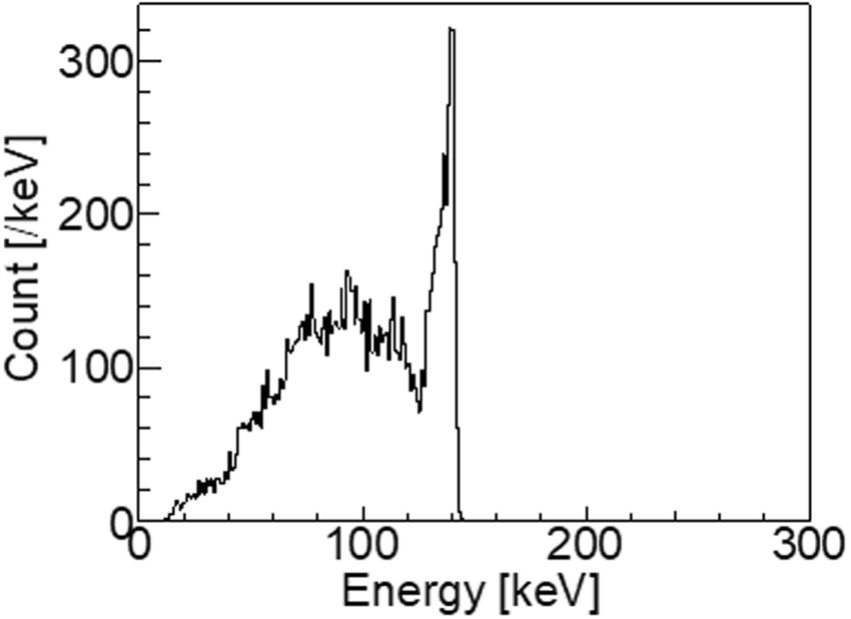
Energy spectrum obtained from simulation.

Figure 1 illustrates the energy spectrum of the simulation study at the activity ratio between the region in the kidneys and that in the body part K/(K+B) = 0.5. From the simulation calculation, 1039 Compton events were selected using the proposed imaging method. Of these events, 650 were from the kidneys and 389 were from the body trunk part.

**Figure 2:**
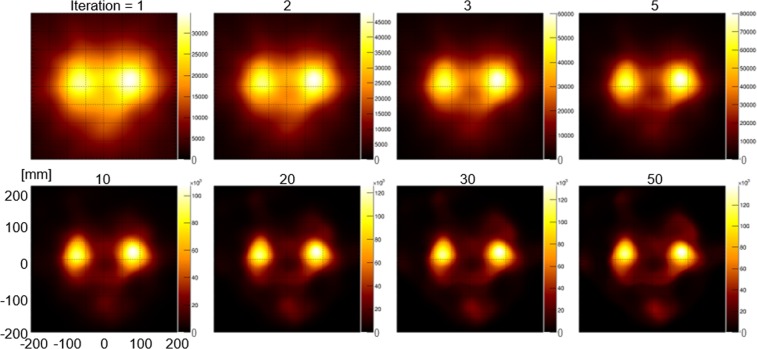
Compton images from simulations with 1 to 50 iterations. The numbers above the images represent the number of iterations.

Using the selected events, Compton images were reconstructed using the list-mode median root prior expectation-maximization (LM-MRP-EM) algorithm with 1 to 50 iterations (Fig. 2). Two bright points representing the kidneys were observed in the reconstructed images. The images were not distinctly different from each other when more than 10 iterations were performed. Therefore, we selected 20 iterations.

**Figure 3:**
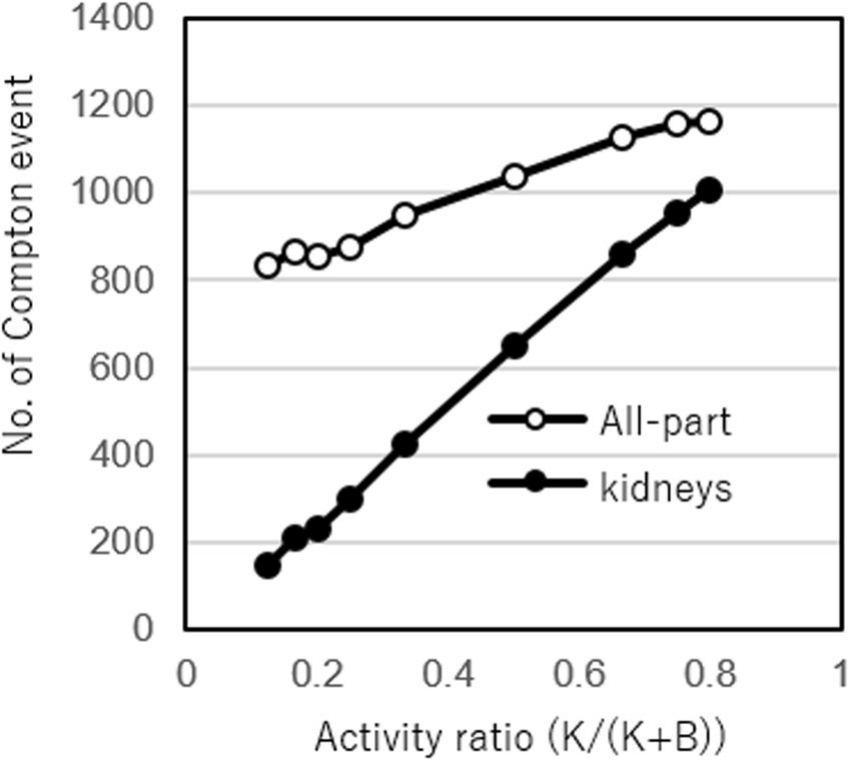
Number of selected Compton events with respect to activity ratio. Open circles represent the number of selected Compton events from the kidneys part and closed circles represent the number of total Compton events (from both the kidney and body parts).

**Figure 4:**
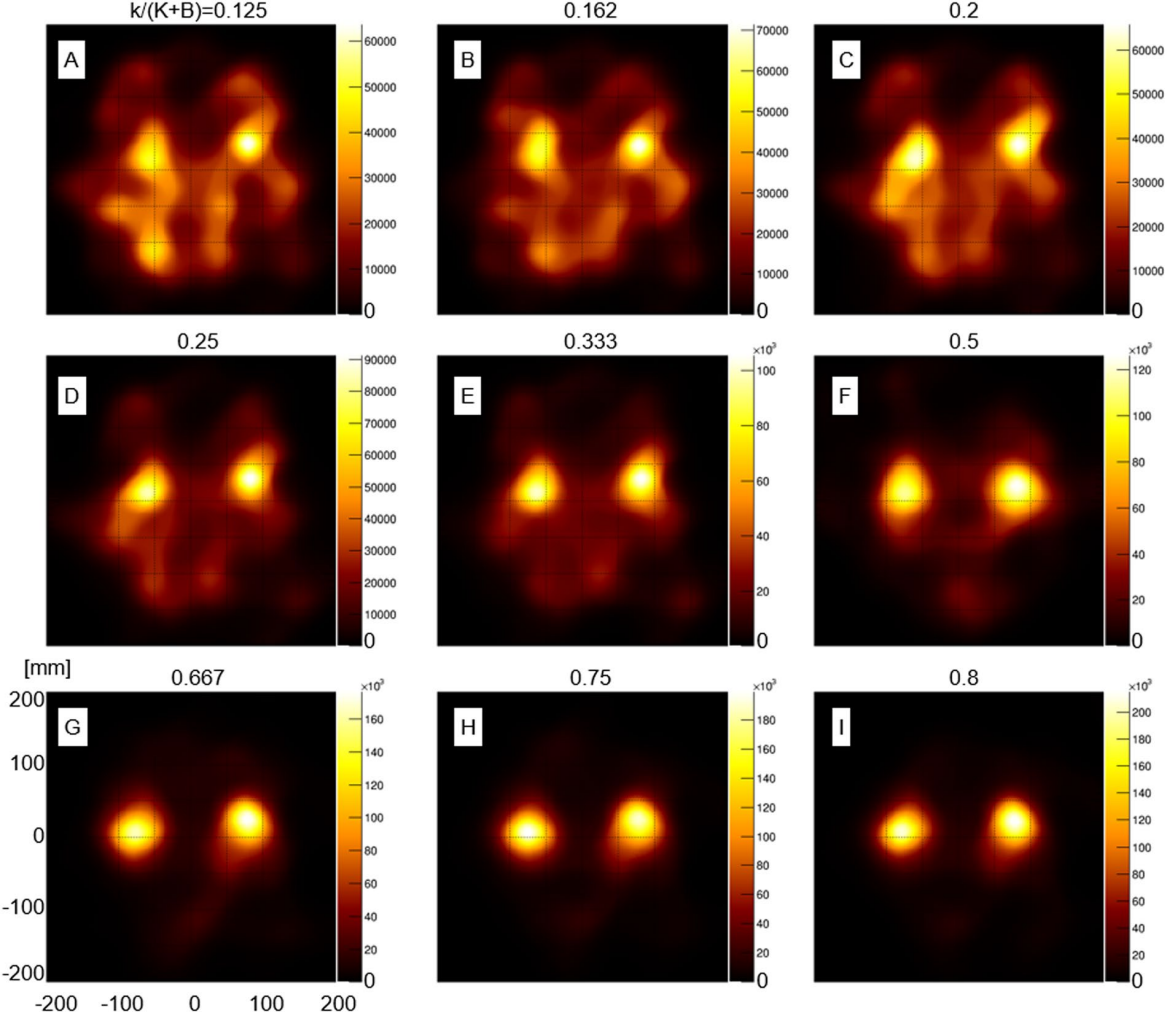
Reconstructed Compton images at K/(K + B) ratio within 0.125–0.8. Numerical values above the images indicate the activity ratio. (**F**) is a re-post of a part of Fig. 1.

**Figure 5:**
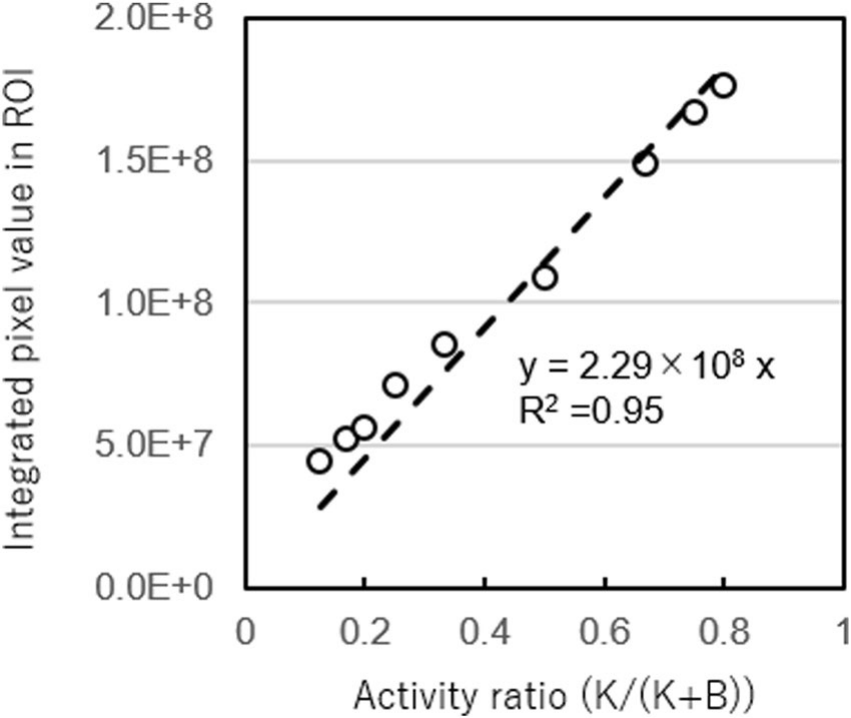
Integral pixel value around the kidney region (dotted circles in Fig. 9(C)).

Figure 3 shows the number of selected Compton events with respect to activity ratio within the kidney (closed circle) as well as within the entire phantom (open circle). Figure 4 shows the reconstructed Compton images with the 20th iteration, when K/(K + B) was within 0.125 to 0.8. For the numerical analysis, the region of interest (ROI) was selected around the periphery of the kidneys. The numerical value in the ROI was calculated by integrating the pixel values within it. The result shows that the integrated value had an increasing trend along with the increasing trend of the activity ratio in the kidney parts (K/(K + B)) (Fig. 5).

### Experimental study

**Figure 6:**
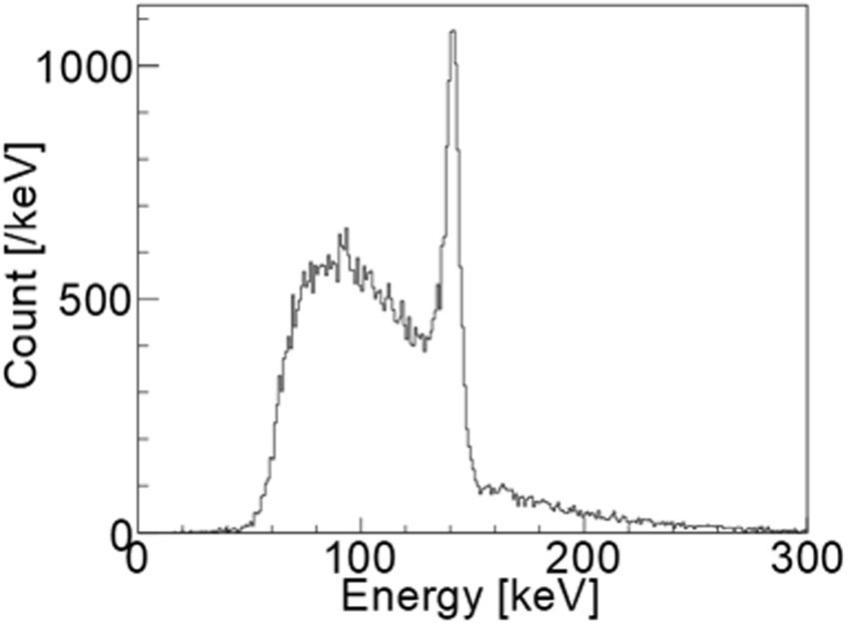
Experimental result of the energy spectrum.

In the experimental study, the live time ratio was 53%. Figure 6 illustrates the energy spectrum of the experimental study. Though, many events were detected with lower energy than 141 keV, the peak was clearly observed at 141 keV. Some events were also observed at energies higher than 141 keV. They would be random coincident events. Using the proposed method, 1205 Compton events were selected.

**Figure 7:**
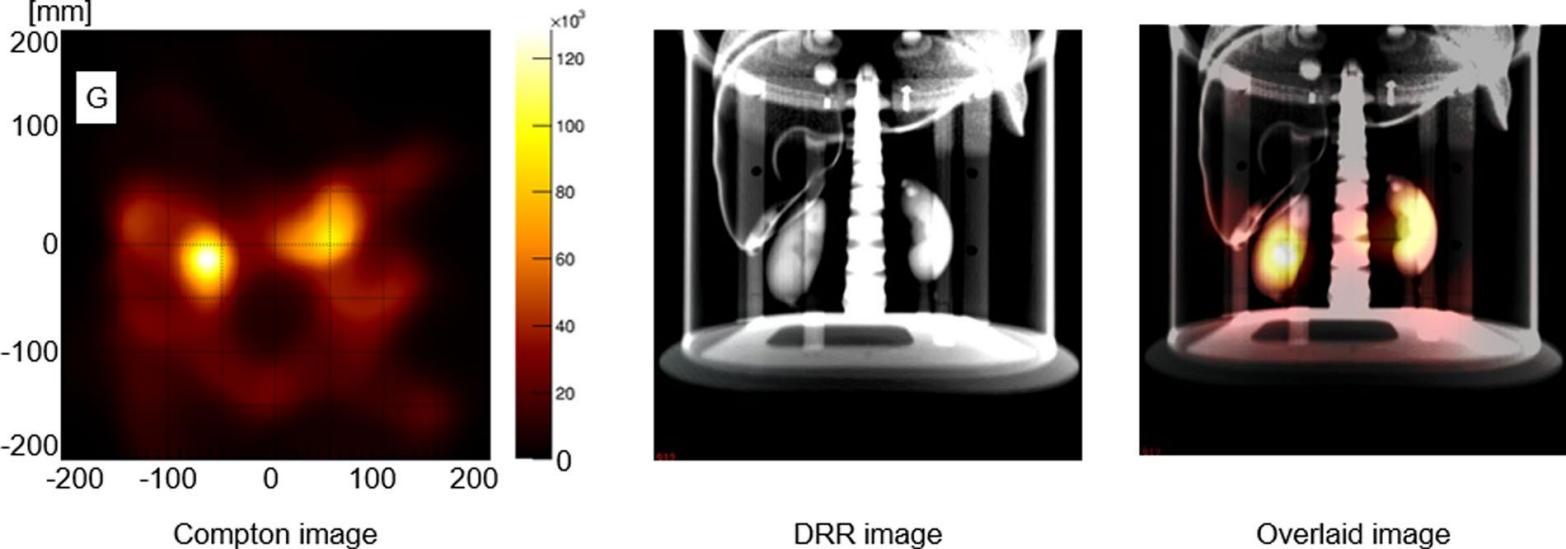
(**A**) Reconstructed Compton image with 20 iterations, (**B**) DRR image of the phantom, and (**C**) Overlay image of (**A**) and (**B**).

Using the selected Compton events, Compton images were reconstructed with the LM-MRP-EM algorithm (Fig. 7) with 20 iterations. The reconstructed Compton image was overlaid with a digitally reconstructed radiograph (DRR) image (Fig. 7C), where the two kidneys could be separately visualised.

## Discussion

In this study, we performed imaging experiments with a human body phantom to confirm the potential of applying a Compton camera to human imaging.

Compton images were reconstructed with the LM-MRP-EM algorithm. The Maximum Likelihood-Expectation Maximization (ML-EM) algorithm can produce quantitatively unbiased images with lower variances than filtered backprojection. However, rules should be defined to stop early iterations because noise tends to increase heavily along the iterations. Therefore, we employed the MRP-EM algorithm, in which a median filter is applied as a Bayesian regularization to control the noise level in the reconstructed images. The median filter is well suited to the reduction of impulse noise, and the MRP-EM algorithm is efficient in stabilizing iterative images generated by ML-EM^18,19^.

In this study, we generated two-dimensional images. The Compton camera determines the incident direction of gamma-rays, and thus, the reconstructed images are affected by the distance of the imaging surface comparing with parallel collimator system. The size of the scatterer is much smaller than the distance, and the error of the distance leads to false enlargement (Supplementary Fig. S1). To generate an accurate image, the correct distance must be known in advance. For example, in static renal scintigraphy, the distance to the kidneys can be easily measured.

In the simulation study, with K/(K + B) = 0.5, LM-MRP-EM could improve images without prominent noises (compared with the ML-EM method (Supplementary Fig. S2). With the 20th iteration, the images showed sufficient convergence and they were not distinctly different from images with more iteration (Fig. 2). Thus, the number of the iteration was fixed at 20 in the other experiments, considering the calculation time. The condition (number of iteration) is acceptable, if the number of Compton events are comparable.

Because numerous individual variations exist, we conducted the simulation study under various conditions. In the images with varying K/(K + B) ratios, the two kidneys were recognizable at activity ratios at least larger than K/(K + B) = 0.2. The number of selected Compton events from the kidney region was proportional to the K/(K + B) ratio. Furthermore, the numerical values in the ROI of the kidneys increased in parallel with the K/(K + B) ratio. The result indicates that the Compton images could be analysed (semi-) quantitatively, although additional investigation is required to verify it. In Compton imaging, the source position of the incident gamma ray is restricted in a cone surface. Thus, the intersection becomes a conic section (ellipse, parabola, or hyperbola). The images were reconstructed by overlapping many conic sections. The complexity of the image reconstruction makes it difficult for analysis in an absolutely quantitative manner^9,20–22^. Therefore, further investigations are required. In addition, for example, the effects of the attenuation and scattering of gamma-rays in the body part of the image were not considered. However, for precise (semi-) quantitative analysis, three-dimensional imaging and corrections for attenuation and scattering would be required.

The result of the simulation with K/(K + B) = 0.5 and that of the experiment were similar. Bright points in the experimental study completely overlapped with the positions of the kidneys. Thus, we believe that our Compton camera can detect gamma rays from kidneys against the effect of attenuation and scattering by body parts in human experiments. The image of the experimental study was slightly indistinct on comparison with the image of the simulation study under the same condition. It may be due to the occurrence of random coincidence events (Fig. 6). Considering the image degradation, K/(K + B) > 0.2 would be required in human imaging experiments.

The sensitivity (detected events/generated gamma rays) was 6 × 10^−8^ in the experimental study, which is much smaller than that of Anger cameras. The sensitivity is greatly affected by the area size of the detector. In actual clinical use, larger sensitivity and better spatial resolution are required. To improve sensitivity, the detector area should be enlarged. The spatial resolution can be improved to 5–6° by spreading the distance between the scatterer and the absorber and setting some scattering angle filter (such as energy window)^14,17^. If the distance from the patient is shortened, both the sensitivity and spatial resolution could be improved. In addition, more information on electron tracking would increase the S/N ratio^23,24^.

As a next step, imaging experiments with a human volunteer is planned. In clinical practice, ^99m^Tc-DMSA is highly deposited in healthy, functioning kidneys. Renal extraction is estimated to be 4–5% per renal passage, and approximately 20–50% of the injected dose is present in the kidneys 1–2 h after injection^25–27^. The numerical value of K/(K + B) within 0.5–0.75 satisfies this condition. Thus, the Compton camera has a potential for imaging the kidneys in human experiments. At the current moment, the imaging ability for 141 keV is not superior to the ordinary scintigraphy cameras. Further investigations are thus required for clinical use. The limitations of this study lie in that activity change over time caused by the accumulation or extraction was not considered. In addition, contrasting density of activity would degrade visibility in human imaging.

## Methods

### Human body phantom

In this study, a human torso phantom (Kyotokagaku, Japan) was used. The phantom contains the kidneys, lower part of a lung, and a backbone in the body trunk part. They were made of acrylic and synthetic bones. The models of the kidneys and liver could store radionuclide solutions, with the kidneys having an inner volume of 100 ml each.

A computed tomography (CT) scan of the phantom was performed by a multi-slice CT system (Aquilion LB, Toshiba Medical Systems) using 2 mm slice thickness. The images were reconstructed using the filtered back projection method.

### Compton camera

**Figure 8:**
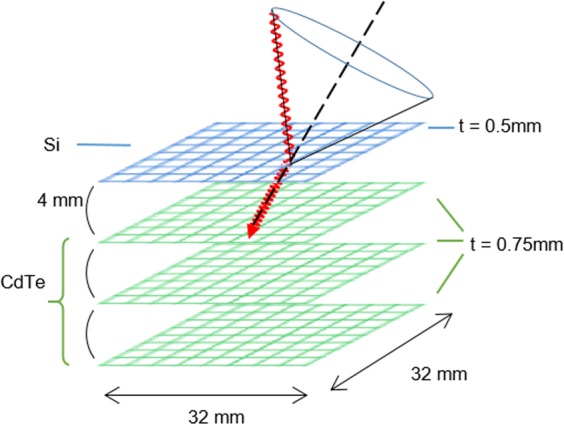
Schematic representation of the Si/CdTe Compton camera. The Compton camera consists of a Si scatterer and three CdTe absorbers.

We used a custom-built Si/CdTe Compton camera (Fig. 8), which consists of one layer of a silicon (Si) detector and three layers of cadmium telluride (CdTe) detectors. The active area of each detector was 32 × 32 mm^2^ divided into 128 strips on each side. The thicknesses of the Si and CdTe detectors were 500 and 750 µm, respectively, and typical energy resolutions (full width at half-maximum) were 2.3 keV at 59.5 keV and 3.8 keV at 81.0 keV, respectively. The detectors and circuits were installed in a chamber and cooled to about −20 °C before operation. The evaluated angular resolution measurement was 9.8° and the evaluated efficiency (selected Compton event/incident gamma rays) was 6 × 10^−4^, for 141 keV^13^. Further details are described elsewhere in previous studies^28^.

### Imaging method

We selected Si-CdTe events, in which energy depositions were detected in the Si detector and one of the CdTe detectors simultaneously. Coincident events detected by both CdTe detectors (CdTe-CdTe event) were discarded. Si-CdTe events concerning the total energy deposition were selected within the 136–146 keV energy range. Si-CdTe events that deposited 20–35 keV energy in the Si detector and the remaining energy in the top layer of CdTe detectors were discarded because such events are typically contaminated by characteristic X-rays of cadmium or tellurium in the topmost CdTe detector^29^.

After the event selection, E_2_ in Equation (1) was replaced by subtracting the initial energy of the gamma rays (141 keV) and E_1_ to improve the angular resolution because the Si detector has better energy resolution than the CdTe detectors. The images were reconstructed with the LM-MRP-EM method^19,30^. Considering geometrical and physical conditions, an efficiency map was calculated analytically^13^. The imaging plane was set at a distance of 270 mm from the first detector in accordance with the distance to the centre of the kidneys. The imaging size was 400 mm × 400 mm, and the pixel size was 2 mm × 2 mm.

### Simulation study

A simulation code was constructed to emulate our Compton camera, and Monte Carlo simulation was performed using the GEANT4 toolkit.

**Figure 9:**
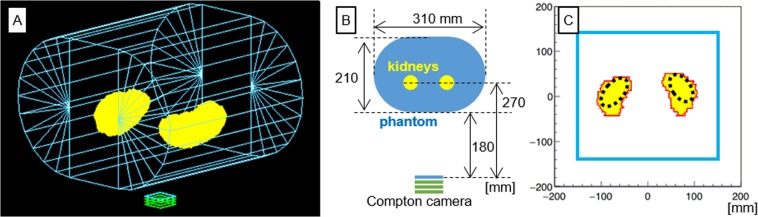
Three-dimensional wired image (**A**), Front image (**B**), Source distribution in the imaging plane (**C**) of the simulation experiment. The wire-frame model represents the body trunk part of the phantom and the Compton camera and the yellow coloured parts represent the kidney parts of the phantom, in (**A**). (**B**) shows the alignment of the phantom to Compton camera for the study. The red and the blue solid lines in (**C**) represent the source distribution of the kidney parts and the body part, respectively. The dotted black line in (**C**) represents the region of interest for evaluating the numerical value of reconstructed image.

A digital phantom of kidneys was designed using the CT data. The part of the body trunk was created with a cuboid (100 mm width, 210 mm height, and 280 mm length) and two semi-circular columns (105 mm radius and 280 mm length) similar to the size of the phantom (Fig. 9). The body phantom was laid in supine position, and the Compton camera was placed under the body phantom at a distance of 180 mm from the back surface of the phantom (Fig. 9B).

Gamma rays of 141 keV—the energy of ^99m^Tc—were isotropically emitted from both the kidneys and the other (body) parts. The number of gamma rays emitted was 2.0 × 10^10^. The activity ratio between the region in the kidneys and that in the body part (K/(K + B)) was changed from 0.125 to 0.8 (K:B ratio was changed from 1:7 to 4:1).

### Experimental study

Each kidney of the phantom was filled with 6.8 MBq of ^99m^Tc solution (FUJIFILM RI Pharma) and the other remaining parts of the body phantom (trunk and liver) were filled with 13.6 MBq of ^99m^Tc solution.

Similar to the simulation, the body phantom was laid in supine position, and the Compton camera was set under the body phantom. The data acquisition time was set to 15 min, from which 1205 Compton events were selected.

The reconstructed image was overlaid with DRR from CT images, which provided a view from the Compton camera reconstructed by a ray tracing method^19^.

## Conclusion

We confirmed the feasibility of a Compton camera for human body imaging through a human body phantom experiment. The kidneys in the phantom were successfully imaged in the experimental study. Considering that the conditions in this study resemble the condition of the typical human body, the Si/CdTe Compton camera has a strong probability of success in human imaging. In addition, our results indicate the capability of (semi-) quantitative analysis using Compton images.

## Supplementary information

Supplementary Figures
