# Supplementary material for: Compton imaging with 99mTc for human imaging
Source: Sci Rep. 2019 Sep 9;9:12906. doi: 10.1038/s41598-019-49130-z (PMC6733951; doi:10.1038/s41598-019-49130-z)
Supplement: Supplementary file 1 — Supplementary Figures [file 41598_2019_49130_MOESM1_ESM.pdf]

Compton imaging with ^{99m}Tc for human imaging

Makoto Sakai, PhD[1)*], Yoshiki Kubota[1)], Raj Kumar Parajuli[1,2)], Mikiko Kikuchi[1)], Kazuo Arakawa[1)], Takashi Nakano, MD, PhD[3)]

[1] Gunma University Heavy Ion Medical Center, Graduate School of Medicine, Gunma University, 3-39-22 Showa-machi, Maebashi, Gunma, Japan
[2] Department of Molecular Imaging and Theranostics, National Institutes for Quantum and Radiological Science and Technology, Anagawa 4-9-1, Inage, Chiba, Japan.
[3] Department of Radiation Oncology, Gunma University Graduate School of Medicine, 3-39-22 Showa-machi, Maebashi, Gunma, Japan

***Corresponding author:**
Makoto Sakai
3-39-22 Showa-machi, Maebashi, Gunma 371-8511, Japan
Tel: +81-27-220-8378
Fax: +81-27-220-8379
E-mail: sakai-m@gunma-u.ac.jp

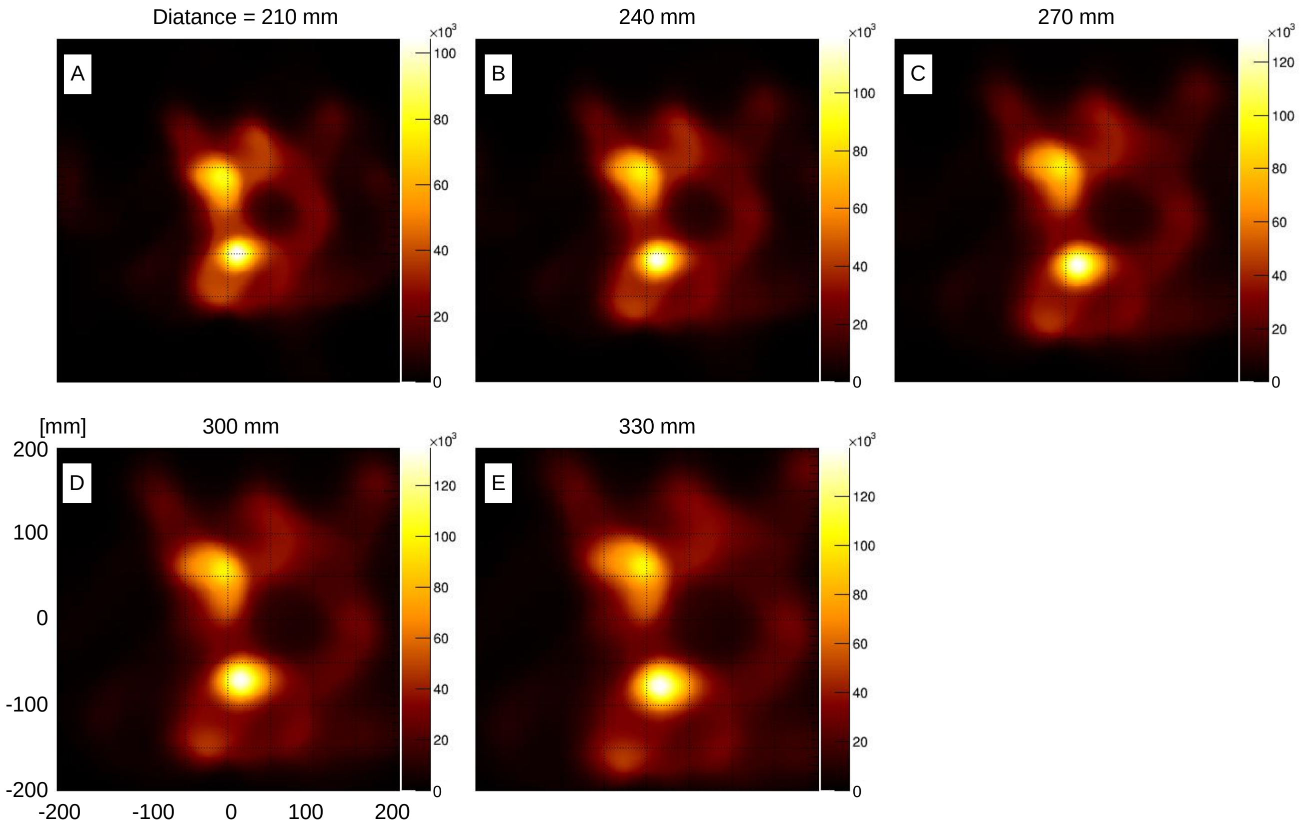

Supplementary Figure S1. Reconstructed images of experimental study with inaccurate distance to the imaging surface. The numerical numbers above the images expressed the distance. (C) is a re-post of Figure 7(A) and it was reconstructed with the correct distance.

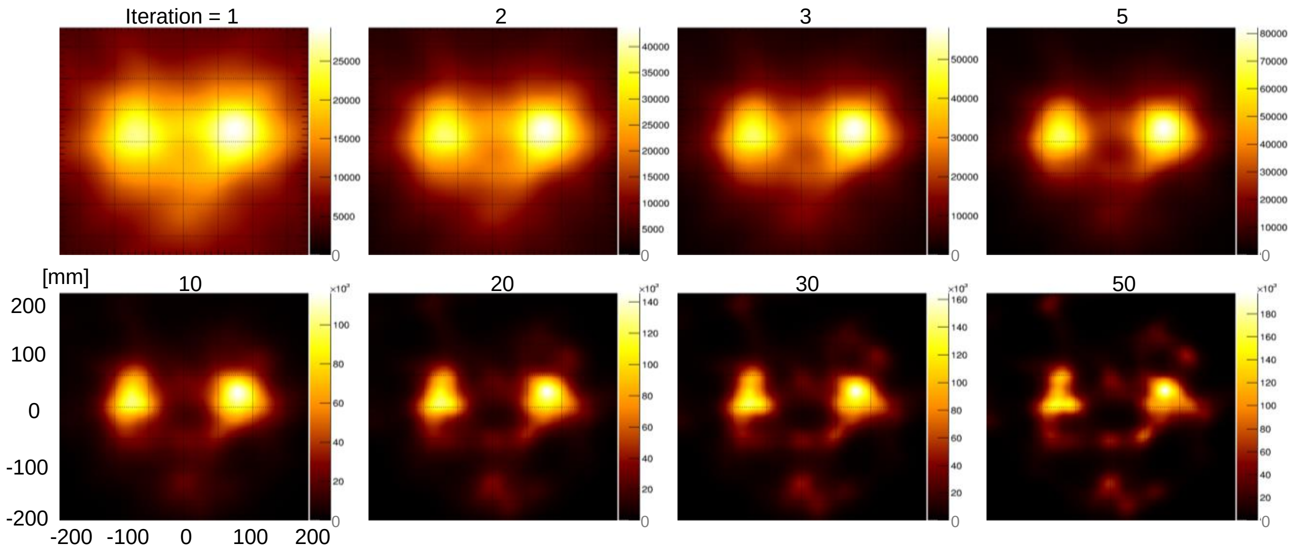

Supplementary Figure S2. Compton images of the simulation study reconstructed using the ML-EM algorithm. The numerical numbers above the images indicated the number of iterations.
